# A comparison of nurses’ and physicians’ perception of cancer treatment burden based on reported adverse events

**DOI:** 10.1186/s12955-019-1210-1

**Published:** 2019-08-22

**Authors:** Shing M. Lee, Jieling Miao, Ruby Wu, Joseph M. Unger, Ken Cheung, Dawn L. Hershman

**Affiliations:** 1 Department of Biostatistics, Mailman School of Public Health, Columbia University, 722 W.168th Street, 6th Floor, New York, NY 10032 USA; 2 SWOG Statistics and Data Management Center, Fred Hutchinson Cancer Research Center, Seattle, WA USA; 3 Department of Medicine, Herbert Irving Comprehensive Cancer Center, Columbia University, New York, NY USA

**Keywords:** Perception of burden, Adverse event burden assessment, Provider consistency

## Abstract

**Background:**

Cancer treatments are associated with a multitude of adverse events (AEs). While both nurses and physicians are involved in patient care delivery and AE assessment, very few studies have examined the differences between nurses’ and physicians’ reporting and perception of AEs. An approach was recently proposed to assess treatment burden based on reported AEs from the physician’s perspective. In this paper, we use this approach to evaluate nurses’ perception of burden, and compare nurses’ and physicians’ assessment of the overall and relative burden of AEs.

**Methods:**

AE records for 334 cancer patients from a randomized clinical trial conducted by the SWOG Cancer Research Network were evaluated by 14 nurses at Columbia University Medical Center. Two nurses were randomly selected to assign a burden score from 0 to 10 based on their impression of the global burden of the captured AEs. These nurses did not interact directly with the patients. Scores were compared to previously obtained physicians scores using paired T-test and Kappa statistic. Severity scores for individual AEs were obtained using mixed-effects models with nurses assessments, and were qualitatively compared to physicians’.

**Results:**

Given the same AEs, nurses’ and physicians’ perception of the burden of AEs differed. While nurses generally perceived the overall burden of AEs to be only slightly worse compared to physicians (mean average VAS score of 5.44 versus 5.14), there was poor agreement in the perception of AEs that were in mild to severe range. The percent agreement for a moderate or worse AE was 64% with a Kappa of 0.34. Nurses also assigned higher severity scores to symptomatic AEs compared to physicians (*p* < 0.05), such as gastrointestinal (4.77 versus 4.14), hemorrhage (5.07 versus 4.14), and pain (5.17 versus 4.14).

**Conclusions:**

These differences in the perception of burden of AEs can lead to different treatment decisions and symptom management strategies. Thus, having provider consistency, training, or a collaborative approach in follow-up care between nurses and physicians is important to ensure continuity in care delivery. Moreover, estimating overall burden from both physicians’ and nurses’ perspective, and comparing them may be useful for deciding when collaborations are warranted.

## Introduction

Understanding the burden of adverse events (AE) resulting from cancer treatments on individuals with cancer is a crucial link in improving care. AEs are any undesirable medical experience associated with the use of a drug, and overwhelming burden as a result of AEs can lead to poor adherence and lack of treatment efficacy. The standard instrument used for capturing and reporting AE is the National Cancer Institute Common Terminology Criteria for Adverse Events (NCI-CTCAE) [1]. The NCI-CTCAE is categorized by system organ classes and each AE is graded on a scale from 1 to 5, with 1 being mild, 2 moderate, 3 severe and interfering with activities of daily living, 4 life-threatening, and 5 death. Conventionally, AEs are captured either by physicians or nurses both in the conduct of clinical trials and in clinic settings. Recently, there has been an effort to evaluate the added value of having patient self-reported symptoms for subjective AEs [2, 3]. However, many questions still remain regarding the best approaches for capturing and assessing the burden of AE on individuals with cancer [4]. Several studies have examined the differences in reporting of AE between patients and clinicians (both nurses and physicians) [5–10]. However, very few studies have examined the differences between nurses’ and physicians’ reporting and perception of AEs.

Two studies have compared nurses and physicians concordance of symptom reporting and grading based on the NCI-CTCAE [11, 12]. Both studies compared individual symptoms captured by nurses versus physicians, and the grading of these symptoms based on the NCI-CTCAE. They found that the capturing of symptoms varies between nurses and physicians, with nurses registering more symptoms compared to physicians [11, 12]. Among the possible reasons cited were differences in symptom attribution with physicians only including AEs associated with treatment, and nurses including symptoms associated with both disease and treatment, differences in the perception of symptom burden on daily of life, and differences in communication between nurses and physicians with patients [11].

Recently, we proposed an approach for estimating a global treatment toxicity burden score using the NCI-CTCAE that incorporates symptoms, as well as, other AEs from the physician perspective [13]. The global treatment toxicity burden is based on the elicitation of overall burden scores using AEs from completed clinical trials, without direct contact with the patients [13]. To evaluate the differences in perception of treatment burden between nurses and physicians, in this paper, we estimate the global burden from the nurses’ perspective using our previously published approach and the same list of AEs from our previous study, thus removing the influence of attribution. This allowed us to evaluate the perception of burden from the nurses’ perspective, and to compare the overall burden and the relative burden for the various organ classes and gradations of AEs between nurses and physicians. Given that differences in the perception of AE burden can lead to different treatment decisions and symptom management strategies, understanding these differences is important because it would suggest the necessity of having provider consistency or collaboration in the assessment and management of AEs.

## Methods

### Patient and data

The adverse event data were captured as part of a multi-center randomized clinical trial conducted by the SWOG Cancer Research Network, a global cancer research community funded by the National Cancer Institute. The study enrolled 746 advanced refractory prostate cancer patients to compare the overall survival of docetaxel and estramustine versus mitoxantrone and prednisone [14]. This trial was selected based on the diversity of the types and grades of AEs observed. Briefly, the median age of the study cohort was 73, 83% were Caucasian and 89% had a baseline Eastern Cooperative Oncology Group performance-status score of 0 or 1. NCI-CTCAE version 2.0 [15] was used to capture AE data, and only maximal grade 3 or higher toxicities that are related to treatment were required to be entered into the database. AEs were captured from 334 out of 746 patients. The remaining 412 patients did not report any grade 3 or higher treatment-related AEs. In addition to the 334 individuals with cancer, 70 duplicate observations were used to ensure internal consistency. These 70 duplicate observations were randomly selected. Each rater was given five identical observations, which were excluded for other analyses.

Using the same elicitation approach previously used with physicians, fifteen nurses at Columbia University Medical Center were enrolled in this study, and each of them was given a list of approximately 50 patients along with their corresponding maximal NCI-CTCAEs and grades [13]. They were asked to mark their burden score based on their overall impression of the combined burden of the adverse events on the patient’s overall health, quality of life and function, using a visual analog scale (VAS) from 0 to 10. The VAS had anchors at 0, 2, 4, 6, 8 and 10 cm for no, mild, moderate, severe, life-threatening AEs, and death, based on the NCI-CTCAE. The measured distance from 0 to their mark was the corresponding VAS score. Each patient was rated by two randomly selected nurses, which resulted in 668 observations (334 × 2). The lists of AE were identical to those previously provided to physicians. The study was approved by the Institutional Review Board at Columbia University Medical Center (CUMC IRB#AAAL7451).

### Statistical analysis

The agreement between duplicate observations within the same rater, and the pairs of observations across the two raters were evaluated using intra-class correlations. For the comparison of the VAS between nurses and physicians, the VAS burden scores from the two raters were averaged to incorporate the information from both raters. The averages were then compared between nurses and physicians using plots, summary statistics and the paired T-test. VAS was also categorized into five categories VAS of 0–2, 2–4, 4–6, 6–8 and 8–10, based on the NCI-CTCAE anchors (0 = No, 2 = Mild, 4 = Moderate, 6 = Severe, 8 = Life-threatening, 10 = Death). The agreement between nurses and physicians was compared by these categories. We also compared the agreement for VAS > 4 (moderate or worse) and VAS > 6 (severe or worse) using Kappa statistics to evaluate where disagreements occurred. The Kappa statistics is a measure of inter-rater agreement with values over 0.75 suggesting excellent agreement, 0.40–0.75 moderate agreement and less than 0.40 poor agreement [16].

The regression approach used to estimate the severity scores for grade 3 and 4 AEs in the various organ classes of the NCI-CTCAE was identical to that previously used for physicians [13]. Data from both raters were used. Briefly, mixed effects models were used with the nurses’ VAS as outcome. The model included 27 covariates as fixed-effects. Twenty-six covariates corresponded to the grade 3 and 4 events in the 13 organ classes with more than five events, and one covariate corresponded to the presence of a grade 3 AE in any of the four organ classes with fewer than 5 events. These four organ classes (coagulation, endocrine, immunology and skin) were collapsed due to the small number of events. The organ classes represent the different types of AEs. For the modeling, 26 patients were excluded; 15 due to death given that death is an anchor in the VAS and 11 because they did not experience grade 3 or 4 AEs. Forward variable selection with an entry criterion of *p* < 0.05 was applied to identify significant covariates and to obtain a final model. The results were validated using a split sample validation approach. The model fitting was performed using the ‘lme4’ package in R [17]. The final estimated severity scores for nurses were qualitatively compared to previously published physician results.

## Results

### Nurses characteristics

All 15 nurses were female and involved in oncology research. One nurse was excluded due to lack of internal consistency. Thus, data from 14 nurses were used for analysis. The median time they had been employed as a research oncology nurse was 2.5 years (range: 2 months to 17 years). Three out of the 14 nurses were nurse practitioners. The median time to complete the VAS burden assessment for approximately 50 individuals with cancer was 25 min (range: 10 to 44 min). Nurses with two or more years of experience as a research nurse completed the VAS burden assessment faster compared to those with one year or less of experience (mean of 18 min versus 35 min).

### Comparison of VAS

For the 334 patients who had a least one AE captured, the mean difference in the average VAS score was 0.30 (95% CI, 0.16, 0.45; *p* < 0.001), with nurses scoring slightly higher (i.e. worse toxicity burden) compared to physicians. While the difference is statistically different, the scores were only slightly worse and may not be clinically relevant. The mean average VAS score for nurses across the two raters was 5.44 (SD = 1.76), compared to 5.14 (SD = 1.92) for physicians. When categorizing the average VAS into 0–2, 2–4, 4–6, 6–8 and 8–10, nurses and physicians agreed on 170 out of the 334 patients (51%). The disagreement between nurses and physicians was more pronounced for mild to severe VAS burden scores (2–6) compared to severe to life-threatening VAS burden scores (> 6). Table 1 displays the cross-tabulation of the categorized VAS for nurses and physicians. The percent agreement for having a VAS of 6 or greater between nurses and physicians was 78% with a Kappa of 0.49. The percent agreement for having a VAS of 4 or greater between nurses and physicians was 64% with a Kappa of 0.34. This suggests poor agreement between nurses and physicians particularly in the mild to severe range of VAS. The intra-class correlation for the five duplicated VAS scores across the 14 nurses was 0.83 compared to 0.91 for physicians. The intra-class correlation for any two pairs of nurses was 0.50 compared to 0.59 for physicians.

**Table 1:** Cross-tabulation of the number of patients assigned to the various categories of Visual Analog Scale (VAS) by Nurses versus Physicians

	Physicians VAS Categories				
Nurses VAS Categories	0–2	2–4	4–6	6–8	8–10
0–2	**4**	4	0	1	0
2–4	5	**25**	19	4	0
4–6	6	45	**89**	23	1
6–8	0	5	37	**35**	8
8–10	0	0	1	5	**17**

The categories of VAS are 0–2, 2–4, 4–6, 6–8 and 8–10. The anchors on the VAS were 0 = No Adverse Event, 2 = Mild, 4 = Moderate, 6 = Severe, 8 = Life-threatening, 10 = Death. The numbers on the diagonal in bold indicate agreement between nurses and physicians in regards to the VAS category. The numbers to the right of the diagonal indicate the number of patients for which physicians assigned higher VAS categories compared to nurses. The numbers to the left of the diagonal indicate the number of patients for which physicians assigned lower VAS categories compared to nurses. Entries in bold indicate the number of patients for which physicians and nurses agreed

### Comparison of severity scores

The estimated severity scores for the presence of grade 3 or 4 AEs are displayed in Table 2 by system organ class for both nurses and physicians. Nurse’s severity scores were generally higher compared to physician’s. Only the severity score for grade 4 genitourinary/renal events was significantly lower for nurses compared to physicians. However, only one grade 4 renal event was reported which explains the instability of the estimate. In addition, a few severity scores were similar or slightly lower for nurses (grade 3 and 4 hematologic events, grade 3 cardiac events, and grade 3 or higher infections).

**Table 2:** Estimated severity scores for the presence of a single Grade 3 or Grade 4 adverse event in the various organ system classes by nurses and physicians. These estimates were obtained from a mixed effects model with the elicited VAS values as the outcome and the organ system classes as the covariates. The range of the elicited VAS is from 0 to 10

	Nurse		Physician^a^	
System Organ Class	Grade3	Grade4	Grade3	Grade4
Blood/Bone Marrow	4 .02*	5.27*	4.14	5.69*
Cardiovascular	4.99*	6.70*	5.02*	6.02*
Constitutional symptoms	4.46	5.94*	4.14	5.16*
Gastrointestinal	4.77*	6.82*	4.14	5.94*
Genitourinary/Renal	4.46	4.46	4.14	7.38*
Hemorrhage	5.07*	5.07*	4.14	4.14
Hepatic	3.62*	6.66*	3.24*	4.14
Infection	4.91*	4.91*	5.01*	5.01*
Metabolic	5.30*	5.30*	4.14	4.14
Musculoskeletal	4.46	4.46	4.14	4.14
Neurological	4.46	7.33*	4.14	6.77*
Pain	5.17*	5.17*	4.14	4.14
Pulmonary	5.12*	5.12*	4.87*	4.87*

^a^Previously published in Lee et al., 2018 [13]. * *p* < 0.05 in the mixed effects model

Nurses considered symptomatic AE such as gastrointestinal, metabolic, hemorrhage and pain significantly more burdensome compared to grade 3 events for other organ classes (0.31 (95% CI (0.05, 0.58), 0.85 (0.23, 1.47), 0.62(0.17, 1.06), 0.72 (0.38, 1.05) in contrast to physicians. Both nurses and physicians rated grade 3 cardiovascular events, infections and pulmonary events significantly more burdensome compared to other organ classes 0.53 (95% CI (0.17, 0.89)), 0.66(0.16, 1.16), 0.46 (0.13, 0.79). Nurses also rated asymptomatic grade 3 AEs such as hepatic and hematologic events significantly less burdensome compared to other system organ classes. In contrast, physicians only rated hepatic events less burdensome. The results from the five repeated split sample validation were also very similar.

### Calculation of toxicity burden score

For a patient with multiple grade 3 or higher AEs, we present an equation from which to calculate a toxicity burden score (TBS) from the nurses’ perspective using the coefficients from the mixed effects model. Table 3 presents the TBS equation for nurses and physicians, as well as, several examples to illustrate how to calculate a TBS using captured NCI-CTCAEs. In the equation, system organ class followed by the number indicates the presence of an AE in that organ with the specified grade. For example, Hematologic 3 indicates the presence of a grade 3 hematologic event. To calculate the TBS for a patient, we add the intercept along with the coefficients for the grade and type of events that they experience. For example, in the first row of the example, the patient experiences a grade 3 diarrhea and a grade 4 anemia. Using the nurses’ equation we have a TBS of 5.58, in contrast, using the physician’s equation we have a TBS of 4.92. It should be noted that the physician equation does not contain grade 3 gastrointestinal events, and thus nothing is added for the grade 3 diarrhea. Examples of AE combinations that yield similar TBS and discrepant TBS between nurses and physicians are provided in Table 3.

**Table 3:**
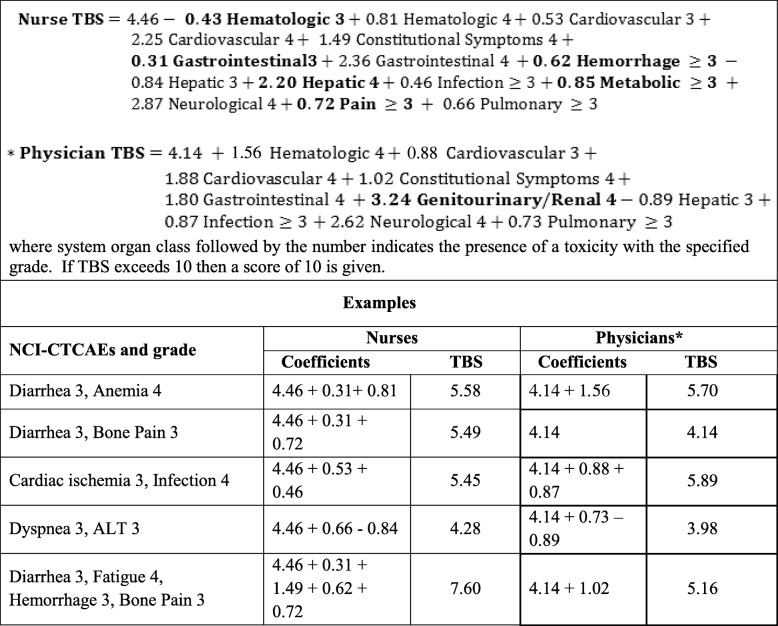
Equation for obtaining a Toxicity Burden Score (TBS) based on nurses and physicians equation and examples of TBS calculations using adverse events captured using the NCI-CTCAE (National Cancer Institute Common Terminology Criteria for Adverse Events)

System organ class that are in bold are only present for either nurses or physicians

^*^Physician equation was previously reported in Lee et al., 2018 [13]

## Discussion

Our results indicate that given the same observed AEs, nurses’ and physicians’ perception of the burden of these AEs differed in several aspects. Nurses generally perceived the overall burden of AEs to be slightly worse compared to physicians with more disagreement in the perception of AEs that were in mild to severe range. Moreover, the perception of the relative burden of grade 3 and 4 events also differed between nurses and physicians. Nurses considered symptomatic AEs to be more burdensome relative to other AEs. This difference in perception may lead to differences in treatment decisions and symptom management, and suggests the importance of having provider consistency, collaboration and cooperation to ensure continuity in care delivery.

This study further supports the need to differentiate the degree of burden between the various organ systems, and to account for the increased burden of the aggregate effect of AEs. Both nurses and physicians considered additional AEs to increase symptom burden. However, AEs are generally reported individually using the NCI-CTCAE making it difficult to assess the overall burden of the AEs in aggregate. Here we propose a simple method for calculating an overall measure of AE burden that accounts for the aggregate effects of adverse events and differentiates between grade 3 and 4 AEs and the various organ classes. This equation, like the NCI-CTCAE, can be utilized across treatment and disease types because the VAS scores solely reflect the toxicity burden. A potential practical application of TBS in patient care is to calculate both nurses and physician TBS, and to implement additional management and care strategy based on the difference in TBS to ensure consistency in symptom management and cancer care delivery. Moreover, additional care and strategy may be implement for patients with higher degree of burden.

A limitation of this study is that it lacked the patient’s perspective to be able to compare nurses’ and physicians’ perception of burden relative to the patient’s own assessment. However, this was hindered by the lack of a validated instrument for assessing overall treatment burden directly from patients. Only one instrument has been proposed to date and has not been further validated [18]. More research is needed in this area. With the availability of validated patient-reported overall treatment burden measure, future studies should compare both physician and nurses’ perception to those of patient’s themselves.

Moreover, future evaluation of the approach should include lower grade and duration of AEs given that nurses and physicians’ difference in perception for these may even be more pronounced. This is particularly important in the era of immunotherapies and targeted therapies, which may not necessarily lead to severe AEs. Another limitation is the small number of nurses and physicians from a single institution, which prevented us from being able to evaluate if the differences in perception were due to gender, training or other factors. Further evaluation in a larger multi-center setting is warranted.

## Conclusions

These results suggest some differences in the perception of treatment burden between nurses and physicians, and point to the importance of provider consistency, provider training and education regarding the collection and management of AEs, or collaboration in the management of AEs, which may help improve patient treatment adherence and quality of life. The proposed TBS can be a tool to help decide when collaborations are warranted. Moreover, both nurses and physicians assessment of burden indicate that the degree of AE burden differs by gradation and type of AEs, and that multiple toxicities lead to increase burden. This highlights the need to move beyond the current emphasis on individual AEs, and the development of better overall toxicity burden summary.

## Data Availability

The datasets used and/or analyzed during the current study are available from the corresponding author and SWOG on reasonable request. The authors do not have full control of the data. Part of the data belong to SWOG and were obtained through the submission of a data request to SWOG.

## Abbreviations

AE: Adverse event
NCI-CTCAE: National Cancer Institute Common Terminology Criteria for Adverse Events
TBS: Toxicity burden score
VAS: Visual analog scale
